# Repurposing riluzole as an anti-osteosarcoma agent

**DOI:** 10.3389/fonc.2025.1508819

**Published:** 2025-05-05

**Authors:** Okkeun Jung, Vinagolu K. Rajasekhar, Syeda Maryam Azeem, Shraddha ChandThakuri, Beatrice Norton, John H. Healey, Shahana Mahajan

**Affiliations:** ^1^ Program in Biology, The CUNY Graduate Center, New York, NY, United States; ^2^ Orthopedic Research Laboratory, Department of Surgery, Memorial Sloan-Kettering Cancer Center, New York, NY, United States; ^3^ Department of Medical Laboratory Sciences, Hunter College, City University of New York, New York, NY, United States; ^4^ Department of Chemistry at Hunter College, City University of New York, New York, NY, United States; ^5^ Program in Biochemistry, The CUNY Graduate Center, New York, NY, United States; ^6^ Brain Mind Research Institute, Weill Cornell Medical College, New York, NY, United States

**Keywords:** osteosarcoma, riluzole, reactive oxygen species, MMP2, metastasis, patient-derived xenograft cell lines

## Abstract

We have studied riluzole, a glutamate-release inhibitor, as a novel anti-osteosarcoma agent. YAP (Yes-associated protein) is recruited by *Bax* promoter to stimulate its expression during riluzole-induced apoptosis in the human metastatic osteosarcoma cell line LM7. Given the substantial genetic heterogeneity in osteosarcoma, studies on the efficacy of riluzole in diverse osteosarcomas will be an asset in developing preclinical studies. Toward this goal, we investigated the effects of riluzole on 11 osteosarcoma cell lines derived from primary or metastatic tumors of mouse or human origin and on four independent patient-derived xenograft (PDX) tumor cell lines. We found that most of the osteosarcoma cell lines, including PDX cell lines secrete glutamate and exhibit invasive abilities. Cell growth and invasive ability of all the cell lines and PDX cell lines are inhibited by riluzole. Additionally, riluzole suppresses the activity of matrix metalloprotease-2 (MMP2) in most of the osteosarcoma cell lines (but not the PDX cells). These results suggest that riluzole’s inhibitory effects on osteosarcoma invasion may in part be attributable to the inhibition of MMP2 activity, and that riluzole is potentially an effective agent for inhibiting growth of primary and metastatic osteosarcomas with a wide range of genetic profiles.

## Graphical Abstract

A schematic of the study.

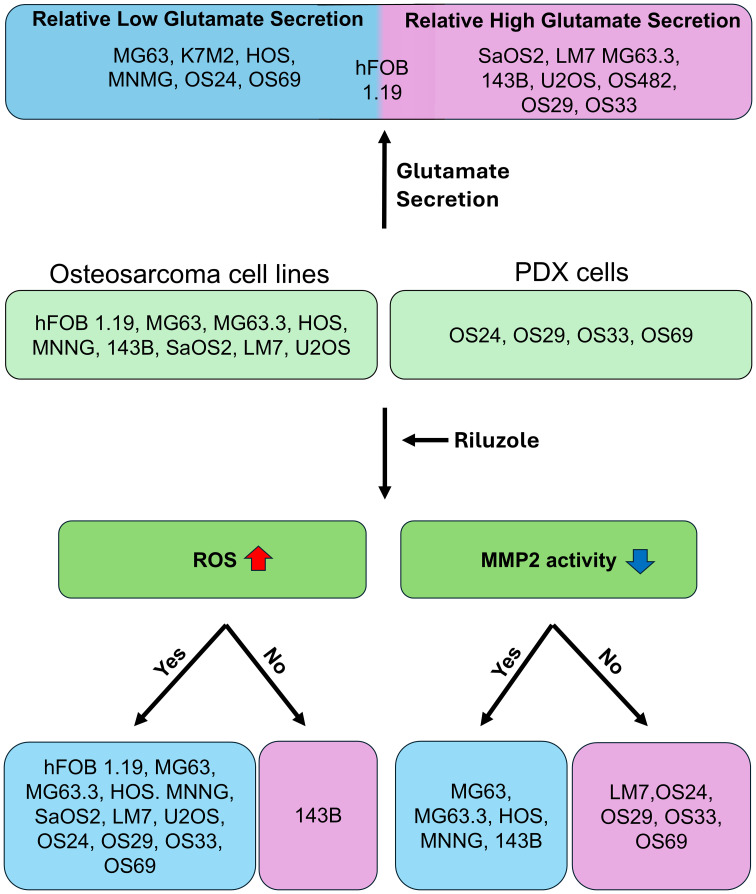

## Introduction

The 5-year survival rate of 30% for patients with metastatic osteosarcoma (OS) has been stable for the past 3 decades, and hence more-effective systemic therapies are urgently needed (1, 2). The need is especially compelling since OS is the third most prevalent cancer in children, adolescents and young adults, and new therapeutic options could greatly extend the life expectancy of these young patients. Systemic spread is typical; 15–20% of patients present with detectable lung and/or bone metastases, and microscopic metastases are present in 85% of patients (3). Despite existing chemotherapies, 40% of treated patients develop local or systemic relapses, only a minority of which can be salvaged (4). One of the major hurdles in the development of an effective OS treatment is the lack of precisely targetable mutations, as tumors have high heterogeneity with complex karyotypes, chromosomal rearrangements, translocations, and deletions (5). Although *p53* mutations are common in OS patients, the mutational status of *p53* is not a clear predictor of detrimental metastasis development (6, 7). Moreover, targeting other commonly seen genetic alterations, including those associated with *Rb*, *CDKN2A*, *PTEN* (phosphatase and tensin homolog), and *Myc*, has been ineffective (8, 9). In addition, the complex and poorly defined microenvironment of OS metastases makes it difficult to treat OS effectively (10). Riluzole, a drug approved for amyotrophic lateral sclerosis (ALS) (11, 12), has been tested in many types of cancers (13). Besides blocking glutamate release, riluzole affects various cellular functions; it modulates glutamate‐dependent and glutamate‐independent intracellular signaling pathways, increases intracellular Ca^2+^, increases oxidative stress, and induces DNA damage in different cancer types (13). Riluzole also arrests the G2/M cell cycle, alters autophagic pathways, and triggers apoptotic pathways (13). As an anticancer drug, riluzole provides advantages due to its low toxicity and its tolerability (14–16). Riluzole has been reported to attenuate Wnt signaling in pancreatic cancer (17) and inhibit proliferation in estrogen receptor–positive breast cancer (18). In a phase II clinical trial, 33% of patients with advanced melanoma who received 100 mg of riluzole twice daily showed downregulation of mitogen-activated protein kinase and PI3K/AKT, and 42% patients achieved stable disease (19). Furthermore, riluzole was well-tolerated; the most common adverse effect was fatigue, experienced by 62% of patients (19). More recently, in a phase I clinical trial, the combination of riluzole and sorafenib was judged safe and tolerable in patients with advanced solid tumors (two of whom had sarcoma) (20). In another trial, the combination of prodrug troriluzole and the anti-PD-1 agent nivolumab resulted in stability of disease in some patients with advanced solid tumors (21). In osteosarcoma, riluzole inhibited release of glutamate (22) and was also shown to inhibit glutamate receptor-dependent cell proliferation (23). In this study, we investigated the efficacy of riluzole in OS across established cell lines and patient-derived models.

Our laboratory has previously demonstrated that riluzole effectively blocks OS proliferation *in vitro* in two cell lines of human (LM7) or mouse (OS482) origin (23). We found that LM7 cells depend on autocrine glutamate signaling for growth and proliferation via signaling through the metabotropic glutamate receptor mGluR5 (23). Subsequently, we showed that riluzole reduces tumor size in a subcutaneous xenograft mouse model (24). Studies in cisplatin-resistant lung cancer and hepatocellular carcinoma found that riluzole increases levels of reactive oxygen species (ROS), thereby inducing cell death (25, 26), and our laboratory similarly found that induction of oxidative stress is the mechanism by which riluzole inhibits proliferation of LM7 (27). Although riluzole has been proven to be effective in two metastatic (mouse and human) OS cell lines (23), its efficacy needs to be demonstrated in other OS cell lines, as well as in patient-derived xenograft (PDX) samples, to establish riluzole as a potential anti-OS drug. Therefore, in the present study, we measured the efficacy of riluzole in 11 primary or metastatic OS cell lines of human or mouse origin, and four primary or metastatic PDX cell lines.

## Materials and methods

### Cell culture

OS cell lines were purchased from ATCC. Specific media requirements have been previously described for LM7 (28), OS482 (9), and MG63 and MG63.3 (29, 30). K7M2 was grown in DMEM with 10% FBS and 1% PS; HOS and HOS-MNNG were grown in EMEM with 10% FBS and 1% PS; U2OS and SaOS2 were grown in McCoy’s 5A Medium with 10% FBS and 1% PS; hFOB 1.19 (hFOB) was grown in DMEM (without phenol red) with 10% FBS and 1% PS; and 143B was grown in DMEM with 10% FBS, 1% PS, and bromodeoxyuridine (0.015 mg/mL).

PDX lines were provided by John Healey’s lab at Memorial Sloan Kettering Cancer Center (MSKCC). OS24, OS33, and OS69 were grown in RPMI with 10% FBS and 1% PS, while OS29 was grown in DMEM with 20% FBS and 1% PS. All cell lines were maintained at 37°C in an incubator supplied with 5% CO_2_.

### Glutamate assay

To measure glutamate release by cultured cells, 6,000 cells/plate were seeded in 3.5-cm plates using glutamine-free media for all cell lines except OS69 and OS33 (for which 12,000 cells/plate were seeded). The media was collected for seven consecutive days, and cell viability was measured using trypan blue staining. At the end of the seventh day, a glutamate assay was performed using a glutamate assay kit from Abcam (#ab83389). In a 96-well reaction plate, 2 µL of the media that had been collected each day was added to wells in duplicate. In each reaction well, 138 µL glutamate assay buffer, 8 µL glutamate developer, and 2 µL glutamate enzyme mix were added and the plate was incubated at 37° C for 30 minutes while protected from light. The absorbance was measured at 450 nm using a spectrophotometer (SpectraMax i3).

### Growth inhibition assay

Growth inhibition was measured using an MTT (3-(4,5-dimethylthiazol-2-yl]-2,5 diphenyl tetrazolium bromide) assay. Twenty thousand cells/well were seeded in 24-well plates for all cell lines except OS69 (40,000 cells/well). After 24 hours, the media was changed and the cells were treated with either dimethylsulfoxide (DMSO), 5 µM riluzole, 10 µM riluzole, 25 µM riluzole, 50µM riluzole or 100 µM riluzole in quadruplicate. The cells were incubated for 48 h at 37°C and MTT reagent was added to cells at 5 mg/mL concentration. The cells were incubated for 2 h at 37°C, and the media was discarded; 1 mL of DMSO was then added to each well to dissolve the precipitated MTT crystals. The plate was incubated for 10 min at room temperature protected from light, and the absorbance was measured by at 570 nm with a reference wavelength at 670 nm using a spectrophotometer. The assay was independently conducted three times for each cell line.

### mRNA quantitation

Quantitative polymerase chain reaction (qPCR) was performed by seeding 0.6 million cells in a 100-mm plate. After 24 h, RNA was extracted following the protocol from the RNeasy Mini Kit (QIAGEN #74106). The RNA concentration was measured using Nanodrop One, and 1 µg of RNA was used for reverse transcription using a QuantiTect Reverse Transcription Kit (QIAGEN #205313). The cDNA was further diluted at 1:10 and used for qPCR. Primers were used at 4 µM concentration. SYBR Green PCR Master Mix (Thermofisher #4312705) was used as the fluorescent probe and GAPDH as the control. The following specific primers were used: Sequences of primers for GAPDH, forward: TGCACCACCAACTGCTTAGC; reverse: GGCATGGACTGTGGTCATGAG. mGluR5, forward: ATGACGGTGAGAGGTCTGCTGA; reverse: GATGCCACCAACAGCTTCTCG. mGluR1, forward: CTGGCATGAAGGAGTGCTGAAC; reverse: GCAGCTCACTTCTCCTTTCCG. Human MMP2, forward: TTGACGGTAAGGACGGACTC; reverse: ACTTGCAGTACTCCCCATCG. Human MMP9, forward: GAGACCGGTGAGCTGGATAG; reverse: TACCGAACGGTGAAGGTGAG.

### Western blotting

For western blotting, we used antibodies for p53 (Proteintech #10442-1-AP), mGluR1 (Abclonal #A11462), mGluR5 (Santa Cruz Biotechnology #sc-293442), vGLUT1 (Abcam #ab272913), xCT (Abcam #ab307601), β-actin anti-mouse 8H10D10 (Cell Signaling Technologies #3700S), and GAPDH anti-rabbit (Cell Signaling Technology #5174S). The secondary antibodies conjugated to horseradish peroxidase were purchased from Cell Signaling Technologies. The western blot signal was detected using C-Digit from LiCOR.

### Reactive oxygen species assay

Cells were seeded in a 24-well plate (40,000–50,000/well). After 24 h, the cells were treated with riluzole (50 or 100 µM) and incubated for 1 h; DMSO was used as control. After 1 h, media from the wells was discarded and PBS containing the fluorogenic probe 2’7’-dichlorodihydrofluorescin diacetate (DCFH-DA) was added to the treated and untreated wells. Next, 50 or 100 µM of H_2_O_2_ dissolved in PBS containing DCFH-DA was added to appropriate wells. The plate was incubated for another hour. Lastly, the cells were washed with 1X PBS and lysed using lysis buffer, and 100 µL was transferred from each well to a flat black clear bottom plate in triplicate. The plate was read at 480 nm/530 nm using a Spectramax i3 plate reader. The DMSO values were subtracted from each of the treated samples. The experiment was repeated three times with triplicate samples.

### Boyden chamber invasion assay

A Transwell membrane (Corning) with 8-µm pole size was used for Boyden chamber invasion assays. The membrane was coated with gelatin-coating solution containing 0.03% gelatin and 0.1% acetic acid for 3 h in a 37°C CO_2_ incubator. The media, containing 1% or 3% FBS plus either DMSO or 25 µM riluzole was placed into lower chamber. Media containing 0.1% FBS was used as a negative control. Cells from OS cell lines and patient-derived lines (U2OS, MG63, MG63.3, SaOS2, LM7 HOS, HOS-MNNG,143B, OS24, OS29, OS33, and OS69) were harvested from the culture plate using trypsin and were resuspended at a density of 4–6 x 10^5^ cells/ml in culture medium containing 0.1% FBS. The media with cells were loaded into the upper chamber at 50 µl/well. This Transwell chamber was placed for 17–20 h at 37°C in a humidified 5% CO_2_ incubator. The membrane with invaded cells was fixed in methanol for 1 h in a −20°C freezer. The invaded cells were then stained using 0.1% crystal in 20% methanol. The non-invaded cells on the upper chamber surface of the Transwell membrane were wiped using wipes soaked in 30% glycerol. The invaded cells were quantified using an optical microscope. The growth inhibition by 25 μM Riluzole was measured for MG63, MG63.3, HOS, MNNG and SaoS2 after 18 hours of treatment to ensure that the growth inhibition was not contributing to the inhibition of invasion.

### Gelatin zymography

OS cells (LM7, MG63, MG63.3, HOS, HOS-MNNG, 143B, OS24, OS29, OS33, and OS69) were treated with either DMSO or 25 µM riluzole in medium containing 0.1% FBS for 48 h. The supernatants were collected and centrifuged to remove cells. After 48 h, the harvested protein content in the conditioned media were quantified using a Bradford assay. Next, the media was mixed with 5X nonreducing buffer and loaded onto an 8% SDS-PAGE gel containing 0.1% gelatin. After electrophoresis, the gels were washed three times with a wash buffer (2.5% Triton X-100 in distilled water). The gels were incubated with a developing buffer containing 50 mM tris(hydroxymethyl)aminomethane and 5 mM CaCl_2_ at 37°C for 48 h in a shaking incubator. Subsequently, the gels were stained with Coomassie blue staining buffer, and the unstained regions were quantified using the ImageJ program to examine MMP2 activity.

### Statistical analyses

The mRNA and protein quantifications in the cell lines and PDX were analyzed by one-way analysis of variance (ANOVA), followed by Dunnett’s test. MTT and glutamate assays were independently repeated three times, and statistical significance was determined using one-way ANOVA followed by Dunnett’s *post hoc* analysis. ROS assays were performed using three biological replicates, and the results were statistically examined by one-way ANOVA followed by Dunnett’s *post hoc* analysis. Invasion assays and gelatin zymography were conducted with three biological replicates, and the results were statistically examined by t-tests. Graphs were created using Prism 9.

## Results

### OS cell lines and PDX cell lines secrete glutamate

We selected 11 cell lines with low or high metastatic potential (**Table 1**), hFOB, 8 human OS lines (SaOS2, LM7, U2OS, MG63, MG63.3, HOS, HOS-143B, HOS-MNNG), and 2 murine OS lines (OS482 and K7M2). The metastatic potential of these cell lines has been evaluated by other labs (31, 32). The lines varied in their mutational status with respect to *p53*, *RB*, *CDKN2A*, and *Myc* expression. Genetic analysis of the PDX cells using the MSK-IMPACT (Integrated Mutation Profiling of Actionable Cancer Targets) tumor-profiling test revealed mutations in *p53* and/or amplifications in other genes (**Table 1**). Specifically, OS24 carries a *EIF5-TP53* fusion gene, an X361_splice in *NCOR1* (nuclear receptor corepressor 1) that results in a truncated protein, a mutation in *DOT1L* (histone methyltransferase) at K97R, and a point mutation in *FGFR3* (fibroblast growth factor receptor 3) at K501M. OS29 has amplifications of the genes for nibrin (*NBN1*), which is involved in DNA repair; cyclin D3 (*CCND3*); vascular endothelial growth factor A (*VEGFA*), which is involved in angiogenesis; and notch receptor 4 (*NOTCH4*). OS33 has a deletion of *RASA1*, a negative regulator of Ras activity which may lead to constitutive Ras activity in these cells. OS69 has a missense point mutation at R337C in *p53*, which is important for oligomerization of p53; this germline mutation is seen in Li Fraumeni and related syndromes, which predispose patients to developing OS (33, 34).

**Table 1:** Characteristics of OS cell lines and PDX cells, including the mutational status of *p53* and other genes.

OS or PDX Cell Line	Organism	TP53 Status (Expression)	*Myc* Level	Metastasis	Other Characteristics
Osteosarcoma					
hFOB	Human	Wild type	—	No	Immortalized fetal normal osteoblast
SaOS-2	Human	Null	Low	Yes	Mature osteoblast, mutant *Rb* (truncated C-terminus in the Rb protein)
LM7	Human	Null	—	Yes	Mature osteoblast, mutant *Rb*
U2OS	Human	Wild type	High	No	Wild type *Rb*
MG63	Human	Null	High	No	Immature osteoblast, *CDKN2A* null
MG63.3	Human	Null	—	Yes	Immature osteoblast, *CDKN2A* null
HOS	Human	(High)	High	No	*p53* mutation p.Arg156Pro (c.467G>C)
HOS-143b	Human	(High)	High	Yes	Osteolytic, wild type *Rb*, KRAS mutations (p.Ala59Thr [c.175G>A]; p.Gly12Ser [c.34G>A]), *p53* mutation (p.Arg156Pro [c.467G>C])
HOS-MNNG	Human	(High)	High	Yes	Osteolytic, wild type *Rb*, *p53* mutation (p.Arg156Pro [c.467G>C])
OS482	Mouse	Null	—	Yes	Osteolytic
K7M2	Mouse	Null	—	Yes	—
PDX					
OS24	Human	EIF5-TP53 oncogenic fusion	ND	Yes	Additional mutations in *NCOR1* (X361_splice), *DOT1L* (K97R), and *FGFR3* (K501M)
OS29	Human	Wild type	ND	No	Amplifications of *NBN1*, *CCND3*, angiogenesis (*VEGFA*), and NOTCH4 genes
OS33	Human	Wild type	ND	No	Genes for GTPase-activating protein (GAP) and negative regulator of RAS (*RASA1*) are deleted
OS69	Human	TP53 (R337C)	ND	No	Missense mutation in *p53* (R337C), a tumor suppressor in the DNA damage pathway

OS, Osteosarcoma; PDX, Patient-derived xenograft; ND, not determined.

**Figure 1:**
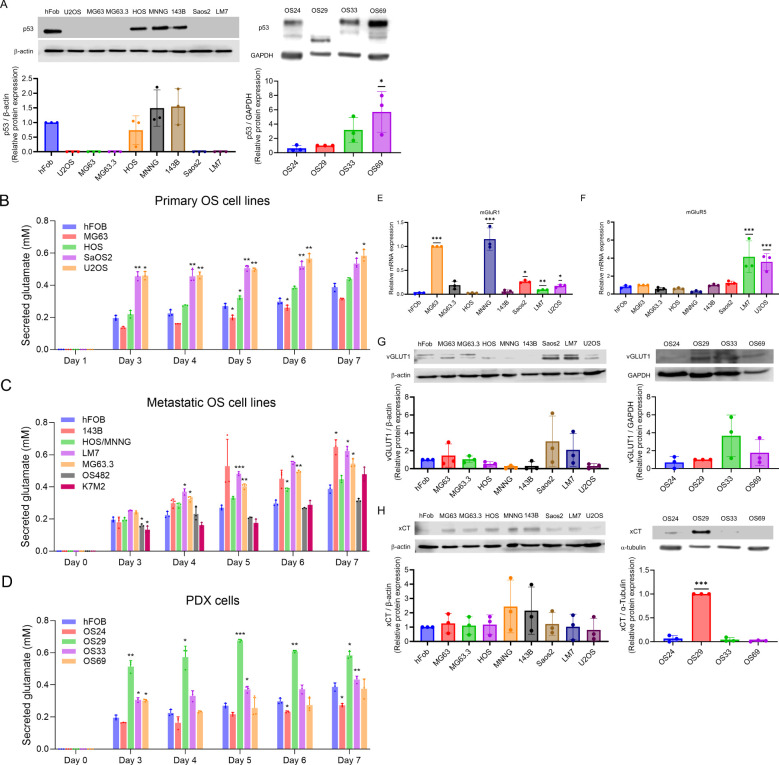
p53 expression and glutamate secretion in OS cells. **(A)** Western blots showing expression of p53 in OS cell lines and PDX cells. The data was analyzed by one-way ANOVA followed by Dunnett’s test. *p<0.05. **(B–D)** Glutamate secreted into media between days 0 and 7 by primary OS cell lines, hFOB and metastatic cell lines, and hFOB and PDX cells. The experiment was repeated three times; the average of the three is shown. The data was analyzed by one-way ANOVA followed by Dunnett’s test for each day, with the value for hFOB for that day serving as the comparison value. *p <0.05, **p<0.01, ***p<0.001. **(E–F)** mRNA expression of mGluR1 and mGluR5 by OS cell lines, measured by quantitative PCR. *p<0.05, **p<0.01, ***p<0.001. **(G–H)** Expression of vGLUT and xCT by OS cell lines and PDX cells, measured by western blot. The data was analyzed by one-way ANOVA followed by Dunnett’s *post hoc* analysis. ***p<0.001.

The clinicopathological characteristics of the patients from whom the PDX lines were derived are shown in **Table 2**. OS24 was isolated from a lung metastasis, while OS29, OS33, and OS69 were isolated from primary bone tumors. Among the OS lines, hFOB had high expression of wild type p53 and HOS, MNNG, and 143B had high expression of mutated p53 (R156P), as previously shown (35), but as expected p53 was not expressed by MG63, MG63.3, SaOS2, or LM7 (28, 36) (**Figure 1A**). Among the PDX cells, OS24, OS33, and OS69 expressed p53, but OS29 did not express a full-length p53 protein (**Figure 1A**). Thus, the cell and PDX lines evaluated in the study carry a variety of mutations, providing the desired heterogeneity for testing the efficacy of riluzole as a treatment for OS.

**Table 2:** Clinical characteristics of patients from whom PDX cells were derived.

PDX Cell Line	Tumor Origin	Site of Isolation	Surgery	Reconstruction	Chemotherapy	Tumor Necrosis	Outcome
OS24	Humerus	Right lower lobe lung wedge	Wide excision	Allograft prosthetic replacement	High-dose methotrexate, doxorubicin, cisplatinum	5%	Dead of disease
OS29	Femur	Right femur lesion	Wide excision	Total knee replacement	High-dose methotrexate, doxorubicin, cisplatinum	98%	Continuously disease-free
OS33	Femur	Right posterior thigh mass	Wide excision	Total knee replacement	High-dose methotrexate, doxorubicin, ifosfamide	20%	Dead of disease
OS69	Femur	Autopsy of bone tissue	Wide excision	Total knee replacement	High-dose methotrexate, doxorubicin, cisplatinum, ifosfamide, etoposide	60%	Dead of disease

Autocrine glutamate signaling may play an important role in the proliferation of OS, and two OS cell lines, LM7 and MG63, have been shown to secrete glutamate (23, 37). To determine if glutamate secretion is common among OS cells, we measured secreted glutamate over seven days in culture media from the primary human OS cell lines hFOB, MG63, HOS, SaOS2, and U2OS (**Figure 1B**), as well as from the murine line K7M2. The results showed a steady increase in glutamate secretion over seven days, with the highest secretion by K7M2 (0.37 mM) followed by HOS and hFOB. The human metastatic OS cell lines 143B, HOS-MNNG, LM7, and MG63.3, and the mouse metastatic cell line OS482, also showed steady increases in glutamate secretion over seven-day period (**Figure 1C**). Glutamate levels were higher in LM7 (0.624 mM), 143B (0.648 mM), MG63.3 (0.545 mM), and K7M2 (0.478 mM) than in hFOB (0.26 mM). Moreover, the nonmetastatic cell lines, such as MG63, and HOS, secreted less glutamate than the metastatic cell lines, such as LM7, MG63.3, and 143B. Among the PDX lines, glutamate levels in OS29 and OS33 on day 7 were higher to those of hFOB, while the glutamate levels in OS69 were comparable to hFOB and those in OS24 were lowest of all (**Figure 1D**). Since dead cells release glutamate into the media, which may interfere with measurement of glutamate secretion, we performed trypan blue staining and confirmed the viability of the tested cells (data not shown). Overall, our results demonstrated that the primary and metastatic OS cell lines and the PDX cells all secreted glutamate, and that levels were generally highest among the metastatic lines.

Our previous work demonstrated that the proliferation of LM7 depends on signaling through the mGluR5 glutamate receptors (23). Moreover, mGluR1 glutamate receptors have been shown to be involved in proliferation of melanoma cells (38). Therefore, we measured mRNA expression of mGluR1 and mGluR5 in human OS cell lines using qPCR, respectively (**Figures 1E, F**). Compared to hFOB, the cell lines MG63, MNNG, SaOS2, LM7, and U2OS had significantly higher expression of mGluR1 mRNA. Similarly, compared with hFOB, LM7 and U2OS cells had higher expression of mGluR5 mRNA, but SaOS2 (the parental cell line of LM7) did not. In essence, the expression of mGluR1 and mGluR5 differed between the primary parental cell lines and the derived metastatic cell lines.

### OS cell lines express vesicular glutamate transporter and xCT

Export of glutamate into the extracellular space occurs through two routes, one mediated by vesicular glutamate transport and the other through xCT (SLC7A11), a functional component of system Xc- (39). Vesicular glutamate transporter (VGLUT) plays an important role in storage and transport of L-glutamate via exocytosis (40). In osteoclasts, the vesicular transport was demonstrated by Morimoto et al. via the transcytosis of the vesicular glutamate transporter 1 (vGLUT1) (40). xCT exports glutamate and imports cystine, which is required for intracellular glutathione synthesis (41). Cancer cells secrete glutamate via xCT (42), and xCT overexpression contributes to proliferation, metastasis, and drug resistance (43, 44).

We measured the expression of vGLUT1 and xCT in all OS cell lines and PDXs to determine the route of extracellular glutamate in each. We found vGLUT1 protein expression in hFOB, MG63, MG63.3, HOS, SaOS2, LM7, and U2OS cells, but little or no expression in HOS-MNNG and 143B cells (**Figure 1G**). All four PDXs expressed vGLUT, and levels did not differ substantially among them. We observed xCT protein expression in hFOB, MG63, MG63.3, HOS, HOS-MNNG, 143B, SaOS2, LM7, and U2OS cells; among PDX cells, OS24 and especially OS29 expressed xCT (**Figure 1H**). Thus, we observed vGLUT and xCT expression in most of the human OS cell lines, and xCT expression in two of the four PDX cells.

### Riluzole inhibits OS growth in cell lines and PDX cell lines

**Figure 2:**
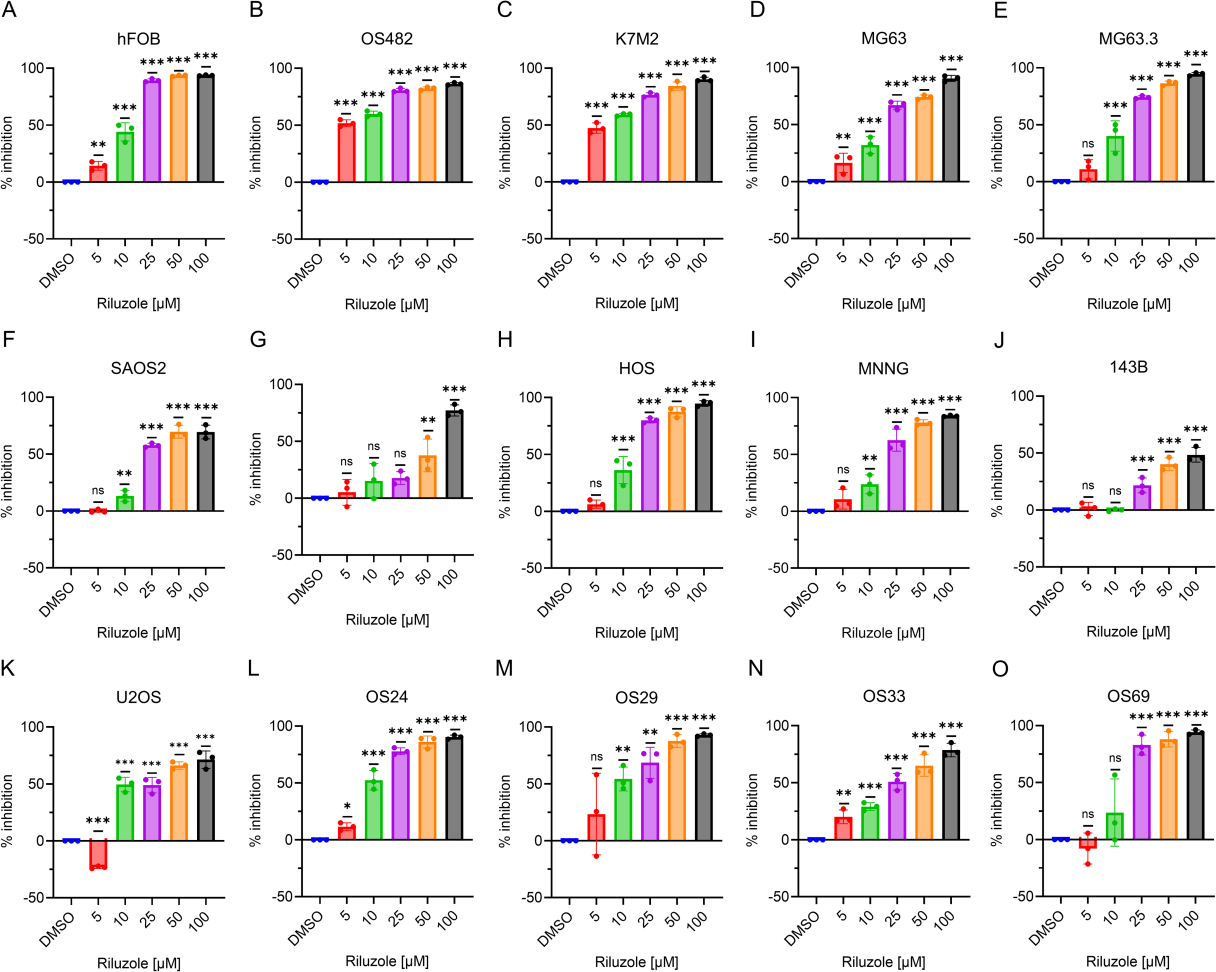
Inhibition of OS cell growth. Percentage inhibition was measured using an MTT assay in all of the OS cell lines using riluzole concentrations of 0, 5, 10, 25, 50, and 100 µM. Cell lines shown are **(A)** hFOB, **(B)** OS482, **(C)** K7M2, **(D)** MG63, **(E)**. MG63.3, **(F)** SaOS2, **(G)** LM7, **(H)** HOS, **(I)** HOS/MNNG, **(J)** 143B, **(K)** U2OS, **(L)** OS24, **(M)** OS29, **(N)** OS33, and **(O)** OS69. Statistical significance was determined by one-way ANOVA followed by Dunnett’s *post hoc* analysis. *p<0.05, **p<0.01, ***p<0.001.

Riluzole is widely used to block glutamate secretion for several neurodegenerative diseases in which excessive secretion is the underlying cause (45, 46). Similarly, riluzole has been tested in many types of cancer that depend on glutamate for growth and proliferation (13); for example, in OS, riluzole blocks proliferation in LM7 and OS482 cells. To investigate the drug’s effect on other OS cell lines, we performed a dose-response growth inhibition assay. We seeded the cells at concentrations designed to ensure that the control cells would not be dead at 72 h and used drug concentrations ranging from 5 µM to 100 µM. The IC_50_ of riluzole varied among cell lines (**Figures 2A–K**). For all cell lines except LM7, there was significant cell death at 25 µM riluzole; for 143B, the IC_50_ remained at 100 µM. We observed proliferation instead of inhibition of U2OS cells at 5 µM, which was consistent across independent experiments. Overall, the data clearly demonstrated that the OS cell lines showed significant, dose-dependent growth inhibition in response to riluzole.

We then tested the patient-derived lines. As **Figures 2L–O** show, the IC_50_ was 10 µM for OS24 and OS29 and 25 µM for OS33. Although OS69 showed variable response at 10 µM, the growth inhibition at 25 µM and above was consistently over 80%. These results demonstrate that the PDX lines consistently respond to riluzole in a dose-dependent manner similar to that of the OS cell lines *in vitro*. The IC_50_ values for riluzole are shown for all cell lines and PDX cells in **Table 3**.

**Table 3:** IC_50_ table of Riluzole in osteosarcoma cell lines and PDX cells.

OS Cell lines	Phenotype	Riluzole IC_50_ (µM)
LM7	Non-metastatic	58.28
SaOS2	Non-metastatic	29.14
U2OS	Metastatic	28.12
MG63	Non-metastatic	17.27
MG63.3	Metastatic	13.79
HOS	Non-metastatic	13.75
143B	Metastatic	89.62
MNNG	Metastatic	20.43
K7M2	Metastatic	7.3
OS482	Metastatic	6.7

PDX Cells	Phenotype	Riluzole IC_50_ (µM)
OS24	Metastatic	11.59
OS29	Non-metastatic	11.08
OS33	Non-metastatic	24.95
OS69	Non-metastatic	15.34

### Riluzole increases ROS in OS cell lines and PDX cell lines except for 143B and OS69

**Figure 3:**
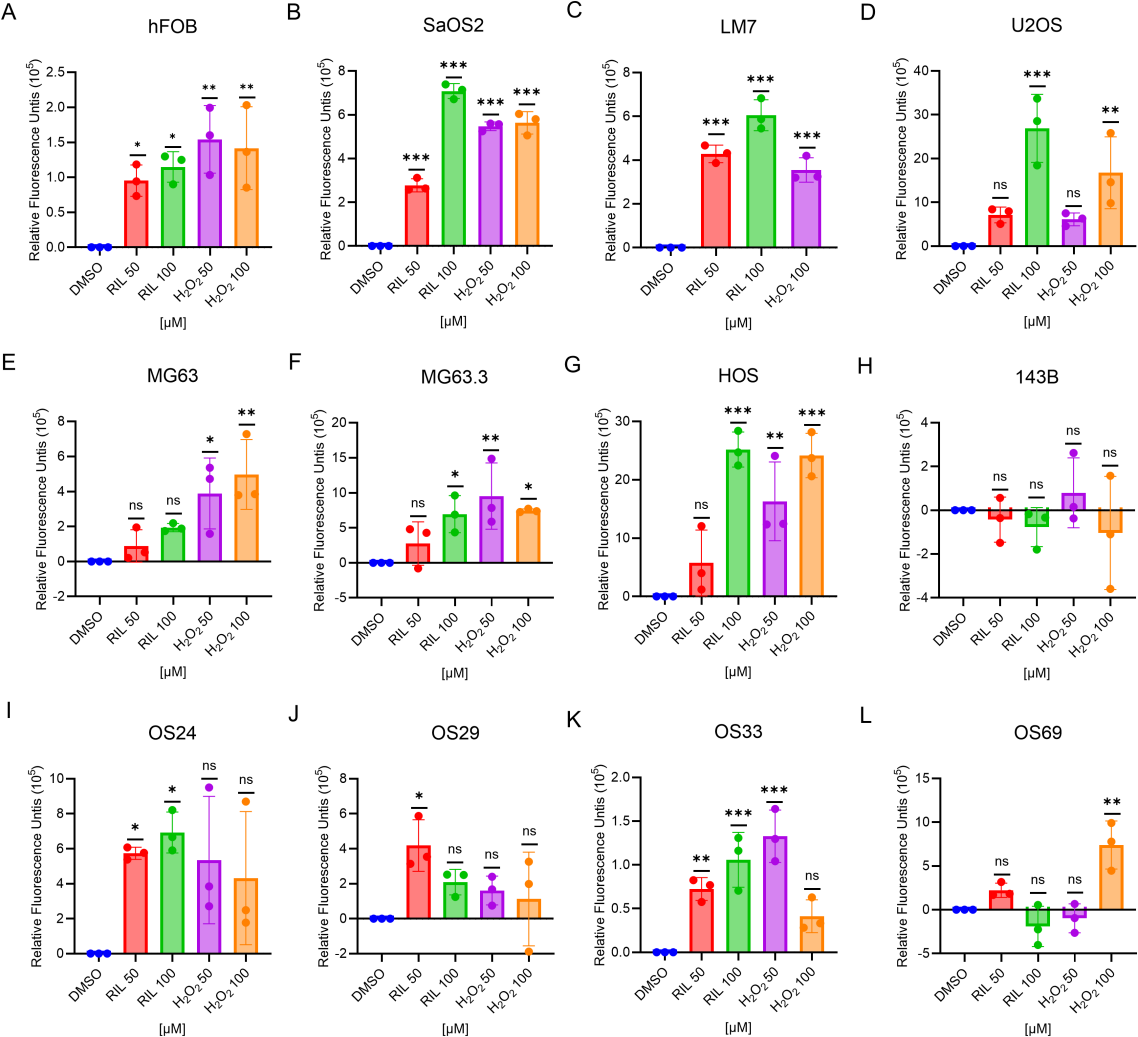
Riluzole treatment increases intracellular ROS except 143B and OS69. ROS production in the cell lines were measured using the DCFH-DA method. The cells were treated with either DMSO (negative control), riluzole 50 µM, riluzole 100 µM, H_2_O_2_ 50 µM, or H_2_O_2_ 100 µM (positive control). Cell lines shown are **(A)** hFOB, **(B)** SaOS2, **(C)** LM7, **(D)** U2OS, **(E)** MG63, **(F)** MG63.3, **(G)** HOS, **(H)** 143B, **(I)** OS24, **(J)** OS29, **(K)** OS33, and **(L)** OS69. The experiments were repeated in triplicates with three different biological samples. Statistical significance was determined using one-way ANOVA followed by Dunnett’s *post hoc* analysis. *p<0.05, **p<0.01, ***p<0.001.

Riluzole inhibits proliferation in hepatocellular carcinoma by increasing ROS (25), and we demonstrated a similar ROS increase in LM7 cells (27). To test if this is a common response to riluzole in OS cell lines and patient-derived cells, we measured ROS production as an indicator of oxidative stress levels. We used riluzole at 50 μM and 100 μM to assess whether any increase in ROS was dose dependent. Of the 8 tested cell lines (hFOB, SaOS2, LM7, U2OS, MG63, MG63.3, HOS, and 143B), all but 143B showed a significant increase in ROS production in response to riluzole (**Figure 3**). We also found significant increases in the four patient-derived lines, though intriguingly in OS69 the increase was apparent at 50 µM but not at 100 µM. Collectively, the data demonstrates that ROS production is a common response to riluzole in OS cell lines and our patient-derived lines.

N-Acetyl-L-Cysteine (NAC) is an established antioxidant agent that has been proved to be effective in compensating for the changes in the ROS (47). To test if cell growth inhibiting by riluzole is due to ROS increase, we performed growth inhibition in the presence of NAC in MG63.3 and LM7 cells. We found the growth inhibition by riluzole was significantly rescued by NAC (**Supplementary Figure S1**).

### Riluzole blocks invasion of OS cell lines and PDX cell lines

**Figure 4:**
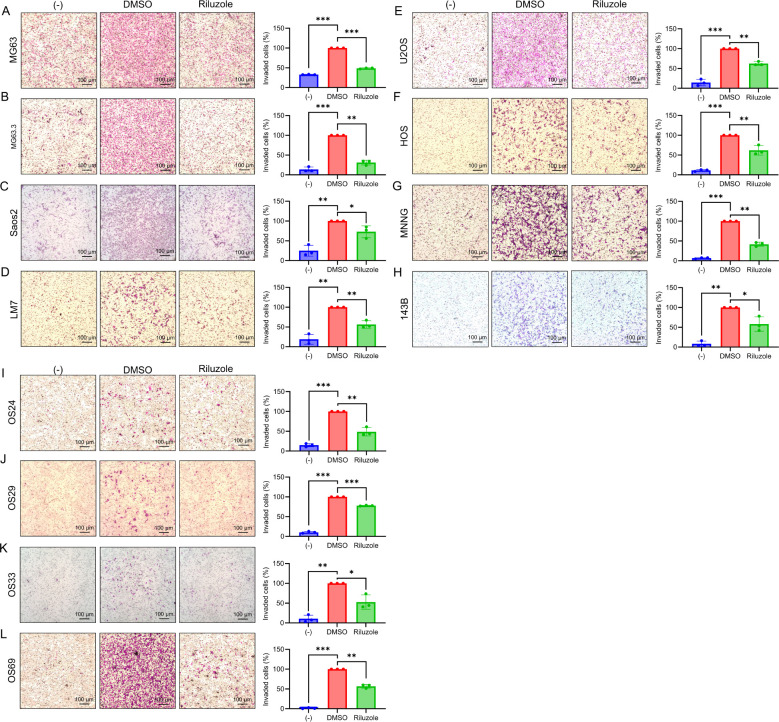
The suppression of invasive abilities in OS cell lines by riluzole. Boyden chamber invasion assay was performed to assess the invasive abilities of OS cell lines. The invasive abilities of **(A)** MG63, **(B)** MG63.3, **(C)** SaOS2, **(D)** LM7, **(E)** U2OS, **(F)** HOS, **(G)** HOS-MNNG, **(H)** 143B, **(I)** OS24, **(J)** OS29, **(K)** OS33, and **(L)** OS69 were examined in the presence of culture media containing 1% FBS. The cells were treated either with DMSO (control) or with riluzole at 25 µM. Media with 0.1% FBS was used as a negative control (−). Magnification bar in the image represents 100 μm. Statistical significance was determined using Student’s t-test. *p<0.05, **p< 0.01, ***p<0.001.

One of the hallmarks of cancer cells is the ability to invade surrounding tissues (48). We previously used a scratch assay to demonstrate that riluzole inhibits migration of LM7 cells (23). Here, to determine the drug’s effects on invasion properties of OS cell lines and patient-derived lines, we measured cell invasion using a Boyden chamber assay in the presence or absence of riluzole. To prevent the interference of cell death in the invasion assay, we used riluzole at a lower dose (25 μM) and set the assay duration to 18 hours. We found significant inhibition of cell invasion in the OS cell lines MG63, MG63.3, SaOS2, LM7, U2OS, HOS, HOS-MNNG, and 143B (**Figures 4A–H**), and in the patient-derived lines OS24, OS29, OS33, and OS69 (**Figures 4I–L**).

### Effect of riluzole on MMP2 activity in OS cell cultures

**Figure 5:**
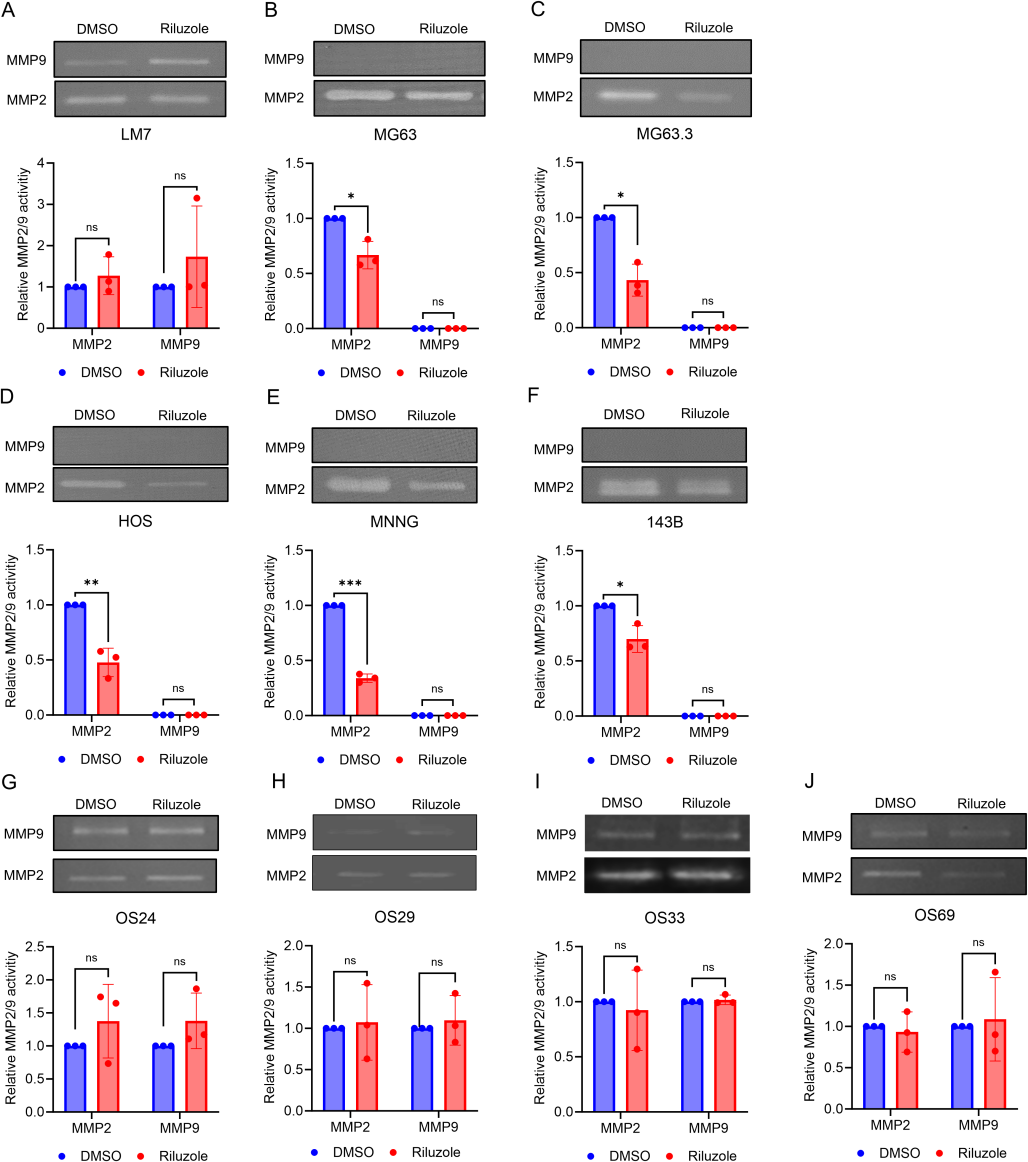
The inhibition of MMP2 activity in OS cell lines by riluzole. Gelatin zymography analysis was used to examine the activity of MMP2 and MMP9 secreted by **(A)** LM7, **(B)** MG63, **(C)** MG63.3, **(D)** HOS, **(E)** HOS-MNNG, **(F)** 143B, **(G)** OS24, **(H)** OS29, **(I)** OS33, and **(J)** OS69 cells. The cells were treated with either DMSO (control) or riluzole at 25 µM. The band intensity was quantified using ImageJ. All experiments were independently repeated three times. Statistical significance was determined using Student’s t-test. *p<0.05, **p<0.01, ***p<0.001.

Matrix metalloproteinases (MMPs), a family of extracellular matrix remodeling enzymes, play an important role in invasion and metastasis of cancer cells by cleaving components of the extracellular matrix (49). Specifically, MMP2 (gelatinase A) and MMP9 (gelatinase B) are associated with tumor spread and cell invasion (50, 51). Because riluzole inhibits the invasion of OS cells, we assessed activity of MMP2 and MMP9 to investigate their potential role in the invasive properties of OS cells. We performed gelatin zymography for OS cell lines and patient-derived cells in the presence or absence of riluzole. We found significant decrease in MMP2 activity in MG63, MG63.3, HOS, HOS-MNNG, and 143B, but not in LM7 cells or the four patient-derived lines (**Figure 5**). Moreover, riluzole did not increase MMP9 activity in any of the OS cell lines (**Figure 5**). Thus, it appears that riluzole inhibits cell invasion via inhibition of MMP2 activity in MG63, MG63.3, HOS, HOS-MNNG, and 143B but through other mechanisms in LM7, OS24, OS29, OS33, and OS69 cells.

**Figure 6:**
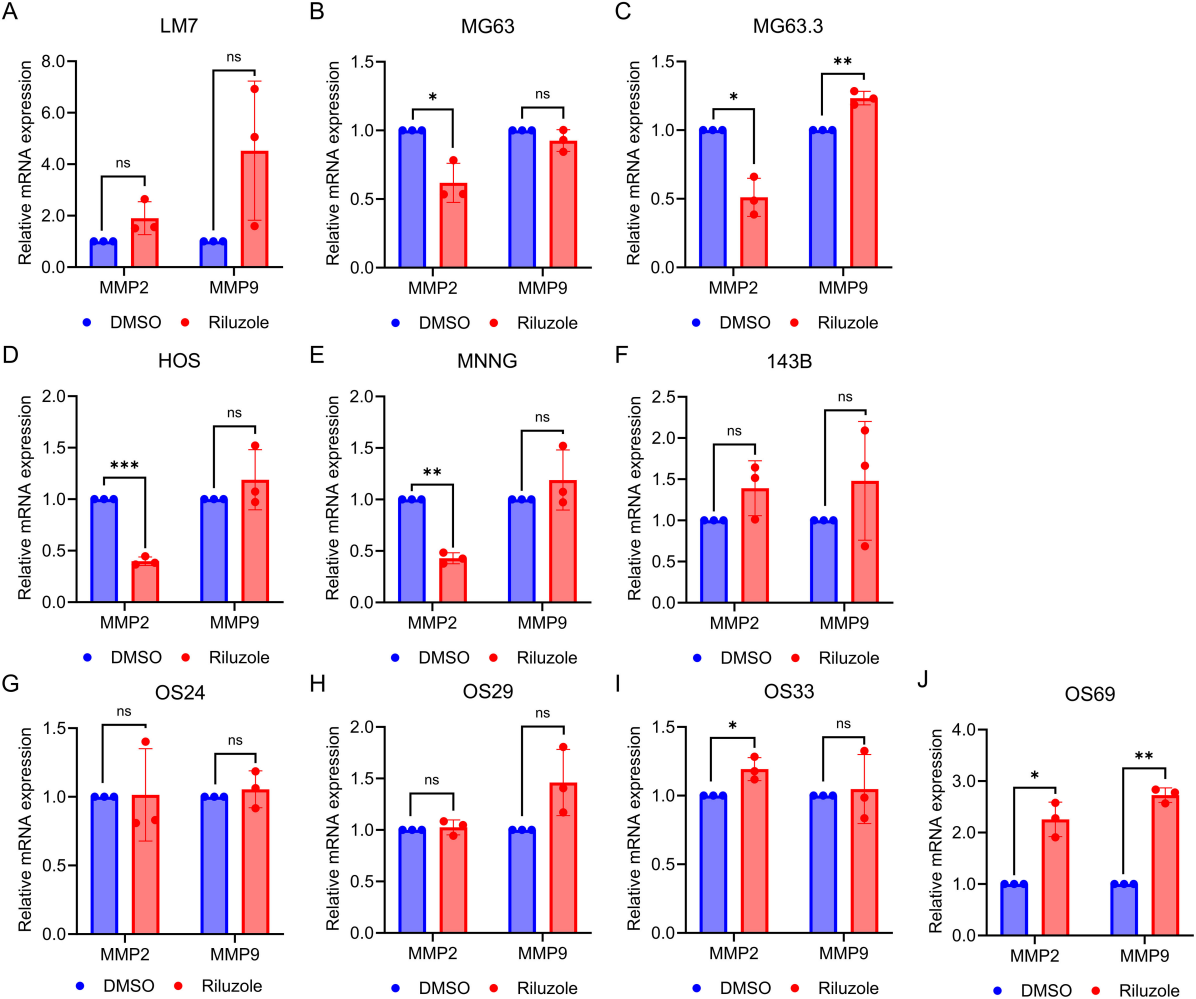
MMP2 activity is transcriptionally regulated by riluzole. Quantitative PCR was performed to measure mRNA levels for MMP2 and MMP9 using total RNA from cells grown in 0.1% FBS and either not treated or treated with 25 μM riluzole for 48 hours. Cell or PDX lines shown are **(A)** LM7, **(B)** MG63, **(C)** MG63.3, **(D)** HOS, **(E)** HOS-MNNG, **(F)** 143B, **(G)** OS24, **(H)** OS29, **(I)** OS33, and **(J)** OS69. Statistical significance was determined using Student’s t-test. *p<0.05, **p<0.01.

To determine if riluzole regulates transcription of MMP2 and MMP9, we measured mRNA levels of these enzymes in the 6 OS and PDX lines. We found that the mRNA levels for MMP2 were significantly reduced in MG63, MG63.3, HOS, and HOS-MNNG cells, but not in LM7 or 143B (**Figure 6**). On the contrary, MMP2 mRNA levels were significantly increased in OS33 and OS69. The only significant changes in MMP9 mRNA expression levels were upregulation in MG63.3 and OS69 (**Figure 6**).

## Discussion

Our data demonstrates the efficacy of riluzole in inhibiting growth and cell invasion in OS cell lines and patient-derived lines of both primary and metastatic origin. The data is consistent with our previous finding that riluzole inhibits cell proliferation in two OS lines, LM7 and OS482 (23). In addition, the present study proves that riluzole is efficacious in OS cell lines of both human and mouse origin and in four PDX lines with distinct mutational profiles, and it suggests that riluzole should be further investigated as an anti-OS agent, as it is efficacious in samples with a variety of mutations.

The data presented here validates the hypothesis that OS cell lines and patient-derived lines secrete glutamate, albeit at varying levels. Levels of glutamate secretion were highest in cell lines that display metastatic behavior and aggressive growth, such as LM7, 143B, and MG63.3, suggesting the potential value of blocking glutamate metabolism. Unexpectedly, OS24, which was derived from metastasized OS in the lung, secreted the lowest levels of glutamate of any of the PDX lines; our future studies will investigate if this characteristic was an acquired adaptation to a distinct microenvironment at the metastatic site. It is not surprising that OS29 cells, which secreted the highest levels of glutamate among the PDXs, overexpress xCT, which may be responsible for the enhanced glutamate secretion.

Riluzole inhibited growth in almost all of the cell lines and PDXs, regardless of the cells’ glutamate secretion level. Moreover, the IC_50_ of riluzole for growth inhibition was generally lower among cell lines or PDXs that secreted lower levels of glutamate. For example, IC_50_ values were particularly low for OS482, whose glutamate secretion levels were one of the lowest among metastatic cell lines, and for OS24, the PDX with the lowest levels of glutamate secretion, suggestion a relationship between secreted glutamate levels and inhibition by riluzole. The higher levels of glutamate secretion among metastatic lines also may be attributable to the higher number of cells in those lines during the assay period. Notably, 143B cells had the highest level of glutamate secretion at day 7, yet they had only 50% growth inhibition when treated with 100 µM riluzole. In contrast, the parental cell line HOS and its sister cell line HOS-MNNG reached IC_50_ at less than 25 µM riluzole. We posit that the resistance of 143B cells to riluzole’s inhibitory effects (as demonstrated by the relatively high IC_50_) may be due to the *KrasG12S* mutation in this line; clinical investigation of riluzole in additional *Kras*-mutant OS cell lines would clarify whether disruption of the pathways regulated by *KrasG12S* contribute to riluzole resistance. Overall, the data cited above suggest the need for a prospective study investigating the roles of glutamate and microenvironment-induced adaptations to glutamate secretion in the tumor cells at the metastasis site in regulating its growth. As the PDX line OS69, which was isolated from a primary tumor site, consistently metastasizes to lung and liver in the animal model (data not shown), it may serve as good model to delineate the mechanism of riluzole in preventing metastasis *in vivo.*

The antiporter xCT, which exports glutamate and imports cysteine into the cells, has been implicated in the proliferation, metastasis, and multidrug resistance of several types of cancer (44, 52, 53) and has been identified as a potential therapeutic target in lung cancer (54, 55). In melanoma, xCT inhibition blocks metastasis (44). Furthermore, riluzole inhibits xCT in breast cancer (56). Because xCT is the source of cysteine for glutathione, a major cellular antioxidant, it plays a crucial role in intracellular redox balance. In glioma, xCT secretes glutamate and contributes to edema and neurodegeneration (57). In our study, OS29 showed elevated expression of xCT while secreting high levels of glutamate. This suggests that the glutamate secretion in OS29 may be occurring via xCT. In essence, riluzole maybe shifting the redox balance to oxidative stress by effectively increasing ROS. Although cancer cells use increased ROS to their proliferative advantage, further increase in ROS is detrimental. High ROS levels initiate a plethora of pathways, leading to DNA damage and eventual cell death by apoptosis. For example, riluzole-induced DNA damage in breast cancer is dependent on the expression of mutant *p53* (14) and Riluzole increases ROS in lung cancer and hepatocellular carcinoma, inducing cell death (25, 26). Our data for all cell lines and PDXs except 143B shows enhanced ROS production upon riluzole treatment. Therefore, increase in ROS by riluzole is an early event leading to inhibition of cell proliferation and cell death, as observed in many cancer types.

Susceptibility to riluzole-induced DNA damage may depend not only on the mutational status of *p53*, but also on the cell’s replicative status, which is dictated by other alterations, one of which may be overexpression of *MYC*. Our previous work has shown that activation of apoptosis is the mechanism of action by which riluzole inhibits proliferation of LM7 cells (27). Riluzole induces ROS-activated cAbl kinase, which phosphorylates YAP at Y357, promoting binding to p73 and, in turn, activating Bax expression (27). In cisplatin-resistant small lung cancer cells, riluzole increases ROS by blocking xCT and further increases ROS by decreasing NAD+, thereby reducing lactate dehydrogenase activity (26). Members of superfamily cytochrome P450 enzymes that metabolize drugs may also contribute to ROS generation, as well as to metastasis and response to chemotherapeutic agents (58). In particular, expression levels of protein isoforms CYP3A4/5 in OS samples predict metastasis and poor survival outcome in patients, as these enzymes dictate the response to chemotherapeutic drugs (59, 60). Whether such metabolic changes contribute to drug resistance in metastatic OS and treatment-refractory patient samples needs to be determined.

In melanoma, riluzole inhibits the invasive ability of cells (61). MMP2 and MMP9 are known to participate in cell invasion and metastasis (50, 51). In our study, cell invasion was blocked by riluzole in OS cell lines and PDX cells, and MMP2 activity was significantly reduced in MG63, MG63.3, HOS, HOS-MNNG and 143B, but not in LM7, OS24, OS29, OS33, or OS69 cells. Moreover, MMP9 activity was not affected by riluzole treatment. Although MMP9 mRNA was significantly increased in MG63.3 and OS69, MMP9 activity did not increase in these cell lines, suggesting that MMP9 expression/activity is controlled post-transcriptionally and that riluzole might impede cell invasion through a mechanism distinct from the inhibition of MMP2 activity in LM7, OS24, OS29, OS33, and OS69 cells. In future studies, it would be valuable to evaluate the effect of riluzole on MMP2 expression in tumor-bearing mice, particularly in cell lines where riluzole has been shown to inhibit MMP2 expression and activity.

A recent study demonstrated that riluzole has analgesic effects in an animal model of prostate cancer bone metastasis (62). Riluzole may also be useful in reducing pain induced by OS, especially osteolytic OS and other cancer types that metastasize to bone and cause bones loss. Therefore, riluzole is a multifaceted drug with multiple effects on cancer, ranging from inhibiting cell proliferation to reducing cancer-induced bone pain.

## Conclusion

As the graphical abstract makes clear, riluzole effectively inhibited growth and invasion *in vitro* in all of the OS cell lines and PDX cells in our study, despite the differences in the mutational profiles of those cells. However, there were differences in the levels of glutamate secretion and in the mechanism of cell invasion among the cell lines and PDXs. Since OS presents with high heterogeneity, it is important to characterize these diverse tumors *in vitro* to assess riluzole’s efficacy across tumors. The differences we observed suggest that riluzole may be effective in a targeted fashion based on the underlying metabolic and genetic profile of an individual cancer. Our present work supports the repurposing of riluzole as a new investigational agent for preclinical studies for OS. The data presented in this study is limited to *in vitro* evaluation. We expect future studies to examine the effect of riluzole in animal models of OS PDXs to pave the way for clinical trials with riluzole treatment in osteosarcoma patients.

## Data Availability

The original contributions presented in the study are included in the article/**Supplementary Material**. Further inquiries can be directed to the corresponding authors.

## Supplementary material

Supplementary Figure 1 Effect of riluzole and N-acetylcysteine (NAC) on cell viability in MG63.3 and LM7 osteosarcoma cell lines. Bar graphs represent the percentage of inhibition in MG63.3 (left) and LM7 (right) cells treated with riluzole alone or in combination with NAC. Cells were treated with either riluzole (25 µM for MG63.3, 50 µM for LM7) or riluzole combined with NAC (5 mM for MG63.3, 10 mM for LM7). Data are presented as mean ± standard error of the mean (SEM). Statistical significance was determined using one-way ANOVA followed by Dunnett’s *post hoc* analysis where *p < 0.05, **p < 0.01, ***p < 0.001.
