# Supplementary figures and images for: Repurposing riluzole as an anti-osteosarcoma agent
Source: Front Oncol. 2025 May 5;15:1508819. doi: 10.3389/fonc.2025.1508819 (PMC12086166; doi:10.3389/fonc.2025.1508819)

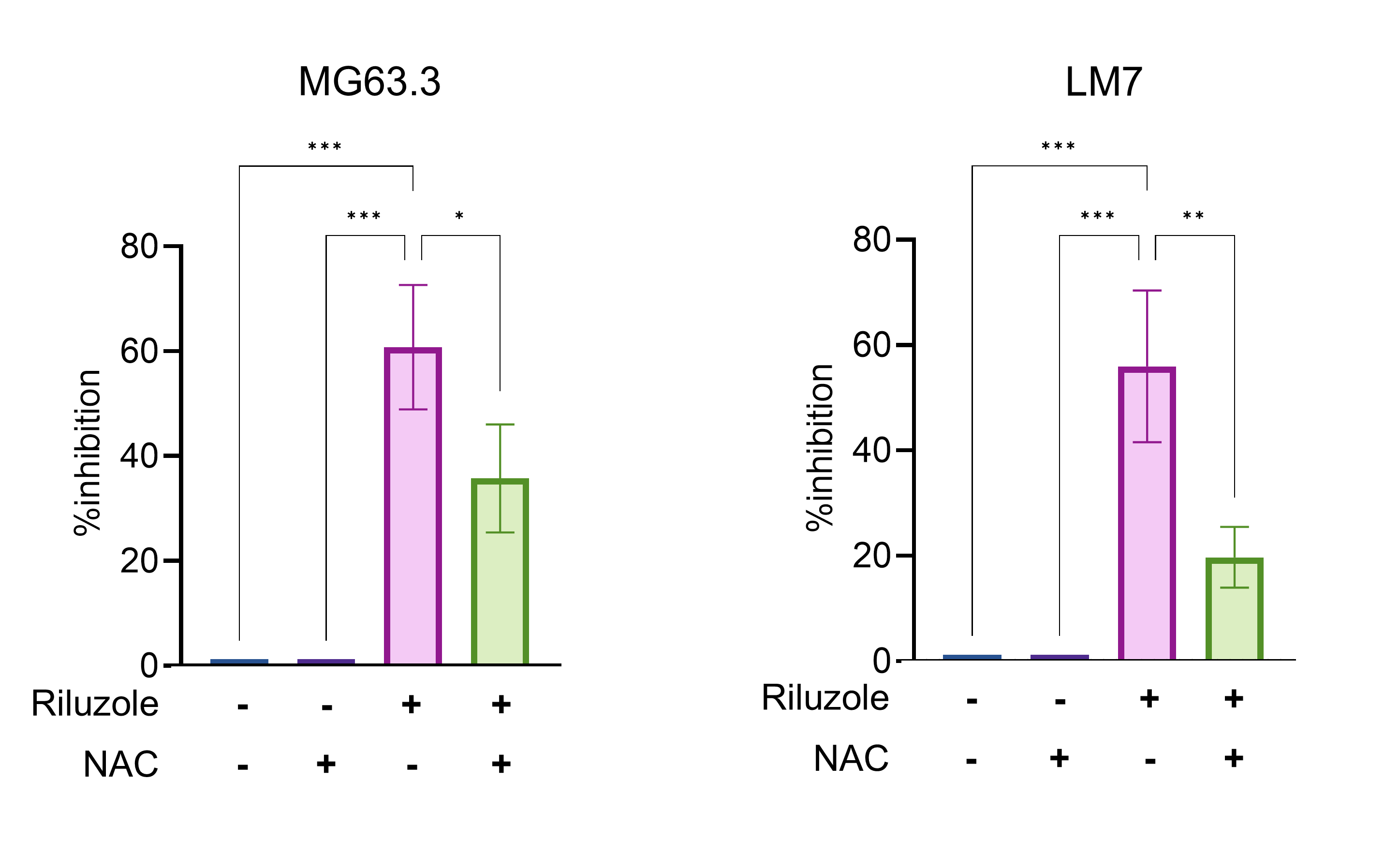

Supplement: Supplementary Figure 1 — Effect of riluzole and N-acetylcysteine (NAC) on cell viability in MG63.3 and LM7 osteosarcoma cell lines. Bar graphs represent the percentage of inhibition in MG63.3 (left) and LM7 (right) cells treated with riluzole alone or in combination with NAC. Cells were treated with either riluzole (25 µM for MG63.3, 50 µM for LM7) or riluzole combined with NAC (5 mM for MG63.3, 10 mM for LM7). Data are presented as mean ± standard error of the mean (SEM). Statistical significance was determined using one-way ANOVA followed by Dunnett’s post hoc analysis where *p < 0.05, **p < 0.01, ***p < 0.001. [file Image1.tif]
